# Fast Acting Insulin Aspart Compared with Insulin Aspart in the Medtronic 670G Hybrid Closed Loop System in Type 1 Diabetes: An Open Label Crossover Study

**DOI:** 10.1089/dia.2020.0500

**Published:** 2021-03-22

**Authors:** Kerem Ozer, Alison M. Cooper, Lily P. Ahn, Cassidy R. Waggonner, Thomas C. Blevins

**Affiliations:** Texas Diabetes and Endocrinology, Austin, Texas, USA.

**Keywords:** Fast acting insulin aspart, Medtronic 670G hybrid closed loop, Type 1 diabetes

## Abstract

This is a single-center randomized open label active-controlled crossover trial comparing efficacy and safety of fast acting insulin aspart (FA) (FIASP^®^) versus insulin aspart (IAsp) (NovoLog^®^) when used in the Medtronic 670G system in auto mode in patients with type 1 diabetes. Forty patients were randomized to either IAsp or FA. Each treatment period was 7 weeks and a standardized meal test was administered 6 weeks after the start of each treatment period. The primary endpoint was postprandial glucose (PPG) increment after the meal test at 1 h. Treatment with FA using the MiniMed 670G hybrid closed loop (HCL) led to a greater reduction in 1-h postprandial glucose increase compared with treatment with IAsp during the standardized mixed meal test. Change in glucose: [estimated treatment difference (ETD ± standard deviation [SD]); 95% confidence interval]: 70.27 (±17.36) mg/dL (3.9 ± 1.0 mmol/L) with FA versus 98.42 (±17.36) mg/dL (5.5 ± 1.0 mmol/L) with IAsp (*P* = 0.008). Patients spent 1.81% (*P* = 0.016) more time (equivalent to 26 min per day) in the 70–180 mg/dL (3.89–9.99 mmol/L) range with FA than with IAsp. The entire sample spent only 0.5% of time <54 mg/dL (<3.0 mmol/L) range. The increment in the 1 h postmeal test glucose was significantly lower with FA versus IAsp. FA in a HCL setting is safe and effective with patients spending more time in the 70–180 mg/dL (3.89–9.99 mmol/L) target range than with IAsp.

Trial registration: Clinicaltrials.gov identifier: NCT03977727.

## Introduction

Hybrid closed-loop (HCL) insulin infusion systems improve glycemic control and reduce hypoglycemia by adjusting basal rates with the input of glucose data from continuous glucose monitoring (CGM) using proprietary algorithms. The Medtronic 670G HCL (670G HCL) system has been shown to be associated with less glycemic variability, more time in target glucose range, low rates of hypoglycemia, and reductions in A1c.^1,2^

One of the challenges of optimizing postprandial glucose control in an HCL system is slower than physiologic absorption of analogue mealtime insulin (insulin lispro, insulin aspart (IAsp), and insulin glulisine).

Fast acting insulin aspart (FA) is a new formulation that contains niacinamide and l-arginine that is responsible for faster initial absorption of IAsp. FA given by subcutaneous (SC) injection has been shown to have greater early glucose-lowering effect than IAsp.^3^ The A1c-lowering effect of mealtime FA has been shown to be noninferior to IAsp in subjects with type 1 diabetes mellitus (T1DM) on multiple daily injection (MDI) regimens.^4^ In a glucose clamp study, FA had a 57% earlier onset of appearance and a 35% earlier time to reach 50% maximum concentration than IAsp.^5^

In a glucose clamp study comparing FA and IAsp given as a bolus on top of a fixed basal rate, the early glucose-lowering effect of FA was twofold higher, onset of glucose-lowering effect was 11 min earlier and offset of glucose-lowering effect was 24 min earlier.^6^ A double-blind crossover study in a non-HCL insulin pump comparing FA with IAsp showed that FA led to significantly greater postprandial glucose (PPG) lowering after a standardized meal test when compared with IAsp.^7^

In 2019, FA was approved for use in the insulin pump on the basis of noninferiority to IAsp in the Onset 5 trial.^8^

This is the first study comparing FA with IAsp in the 670G HCL insulin pump.

This study was done to compare the increment in the 1-h postmeal test using FA and IAsp in the Medtronic 670G system.

CGM-derived time-in-range is a defined and clinically meaningful diabetes outcome measure.^9^ In this trial, we also compared the efficacy of FA and IAsp in the Medtronic 670G system using CGM-measured time-in-range. Safety outcomes were compared as well.

## Materials and Methods

### Study design

This is a single-center randomized open label active-controlled crossover trial with a 2-week run in period and two 7-week treatment periods comparing FA versus IAsp in the Medtronic MiniMed 670G system in participants with T1DM. The study was approved by a central IRB and all patients provided written consent.

### Participants

Adults (>18 years) with T1DM for at least 1 year with an A1c <8.5% and a BMI <35 kg/m^2^ who were using the Medtronic 670G insulin pump and using the same insulin analogue for at least 30 days before screening were eligible. Willingness to remain in auto mode for at least 80% of the time during the study was required. Additional inclusion/exclusion criteria are listed in Supplementary Table S1.

### Insulin trial product

Both FA and IAsp were provided by Novo Nordisk in 10 mL vials (100 U/mL).

### Procedures

After a 2-week screening period, participants were randomized in a 1:1 manner to either open label IAsp or FA. During the screening period, the main focus was to ensure that all participants were familiar with the HCL system, trouble-shooting potential problems, and reviewing study procedures.

In the screening and treatment periods, the investigators targeted preprandial levels of 70–110 mg/dL (3.89–6.11 mmol/L) by adjusting prior meal insulin–carbohydrate ratios. All participants were asked to perform glucose calibration measurements as dictated by the 670G system CGM requirements. Active insulin was standardized to 3 h for all participants throughout the study. The active insulin time used in the Onset 5 study was 3 h. To achieve standardization, we chose 3 h as the active insulin time, which is a commonly used active insulin time.^8^ We determined that the 3 h versus 4 h active insulin time would not have a significant impact on time-in-range data, given that the onset of action and peak is more rapid with FA, and the duration of action is similar.^10,11^ Participants were asked to change their infusion set at least every 3 days.

Participants were instructed to perform 4-point profiles every day during the conduct of the trial (from visit 1 to visit 14) mainly for titration purposes.

The randomized treatment period was 7 weeks after which the participants were crossed over to the comparative treatment. Participants were asked to give their prandial bolus insulin at the start of the meal.

At the end of the treatment period, participants were switched to a suitable approved regimen at the discretion of the investigator and based on their preference.

### Standardized meal test

A standardized meal test (78 g of carbohydrate) was administered 6 weeks after the start of each treatment period. Participants were instructed to bolus immediately before the standardized meal. A standardized liquid meal (two bottles Original Ensure) 440 kcal, macronutrient content: 78 g carbohydrate, 18 g protein, and 12 g fat was served immediately after the bolus dose infusion and consumed by the subject, ideally within 15 min.

Participants were required to be fasting and have self-measured plasma glucose (SMPG) values within a range of 71–180 mg/dL (3.94–9.99 mmol/L) before beginning the meal test and bolus insulin dosing. The participant's body weight was measured and a blood sample drawn 2 min before intake of the standardized liquid meal.

The bolus insulin dose was calculated by the investigator based on the dose level of 0.1 U/kg body weight and was rounded to the nearest whole unit. The 0.1 U/kg dose was used to provide a clinically relevant bolus dose needed for the given size of a standardized meal for T1DM as was done in the Onset 5 study.^8^ The meal was given immediately after the bolus infusion and was to be consumed as quickly as possible (within 15 min). Time points of blood glucose obtained during the meal test were as follows: −2, 30, 60, 120, and 180 min.

### Outcomes

The primary endpoint was PPG increment at 1 h (meal test) after 6 weeks of treatment (i.e., the difference in 1-h PPG and PPG immediately before bolus administration) with FA compared with IAsp when used with the Medtronic 670G system in “auto mode.”

The primary endpoint was analyzed for participants who performed the meal tests in both treatment periods, using the Medtronic 670G system in “auto mode.”

Secondary endpoints that were assessed were the difference in hemoglobin A1c (HbA1c) between periods and glycemic excursion parameters. Glycemic excursion parameters included the following:

(a) Postmeal test 2-h plasma glucose levels at 6 weeks into start of therapy in each arm.
(b) HbA1c, fructosamine, and 1, 5 AG levels before crossover, and at end of study.
(c) Glucose excursion parameters

Time spent (%) within 70–180 mg/dL (3.89–9.99 mmol/L)
Time spent (%) <70 mg/dL (<3.89 mmol/L)
Time spent (%) >200 mg/dL (>11.1 mmol/L)
Hypoglycemia^13^
Severe hypoglycemia

The time spent within 70–180 mg/dL (3.89–9.99 mmol/L), <70 mg/dL (<3.89 mmol/L), and >200 mg/dL (>11.1 mmol/L), and hypoglycemia was defined for each subject as the accumulated time in hours spent within the mentioned intervals as recorded by the CGM component of the Medtronic 670G system.

Comparison between the IAsp and FA groups was analyzed by using linear mixed modeling for repeated measurements for each range. The models included treatment and period as factors, and subject as a random effect.

Other secondary endpoints comparing FA with IAsp in the 670G included the following: total daily dose, change in percentage bolus and basal from baseline, change in insulin: carbohydrate ratio from baseline, percentage time spent in auto and manual modes, infusion site reactions reported by participants, and occlusion events reported by participants.

Fructosamine levels were measured to indicate the average level of blood glucose control over the prior 2–3 weeks. These levels may be a more accurate measure of average blood glucose control after a 6-week treatment period than A1c that measures average glucose control for 2–3 months.

Safety was assessed through hypoglycemia records, patient logs, and HCL parameters.

### Sample size and statistical analysis

It was calculated that there is at least 80% power to detect a treatment difference with 36 subjects. Accounting for 10% dropouts, a total of 40 subjects were randomized. Three subjects had missing follow-up data.

The mean treatment difference stipulated by the estimand (including 95% confidence interval [CI]), alongside treatment means, was derived from a linear mixed model for repeated measurements.

All efficacy and safety endpoints were summarized and analyzed using the full analysis set.

Estimated mean treatment differences (or ratios) were presented together with a two-sided 95% CI for all endpoints analyzed.

Recognizing that there may be more adjustment by the 670G software in the first 2 weeks of treatment in each crossover period, analysis of data was performed for weeks 3 through 7 of each crossover period.

## Results

Overall, 45 patients were screened for participation in the study. Five patients failed screening criteria and three patients had missing follow-up data. These eight patients were excluded from the final analysis. Thus, the final analysis was based on data from 37 patients. Demographics and baseline characteristics were similar between the groups (Table 1).

**Table 1.** Baseline Characteristics (Percentages Where Applicable and Standard Deviations in Parentheses)

Female (%)	32.4 (47.46)	Age (years)	45.7 (12.93)
Male (%)	67.6 (47.46)	BMI (kg/m^2^)	27.1 (3.41)
White (%)	100 (0)	HbA1c (%)	7 (0.54)
Hispanic (%)	5.4 (22.92)	Height (cm)	173.4 (9.29)
Non-Hispanic (%)	94.6 (22.92)	Weight (kg)	81.7 (13.61)

HbA1c, hemoglobin A1c.

Primary endpoint: Glucose increment at 1 h (1 h PPG)

**FIG. 1.**
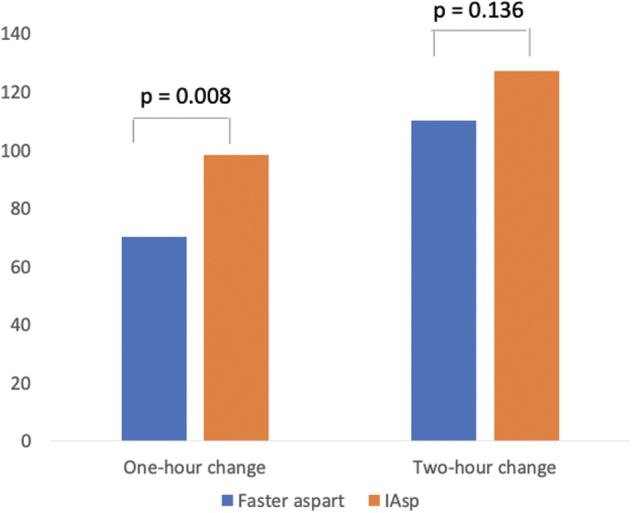
One and 2-h changes during a mixed meal test with faster aspart and IAsp. IAsp, insulin aspart. Color images are available online.

**FIG. 2.**
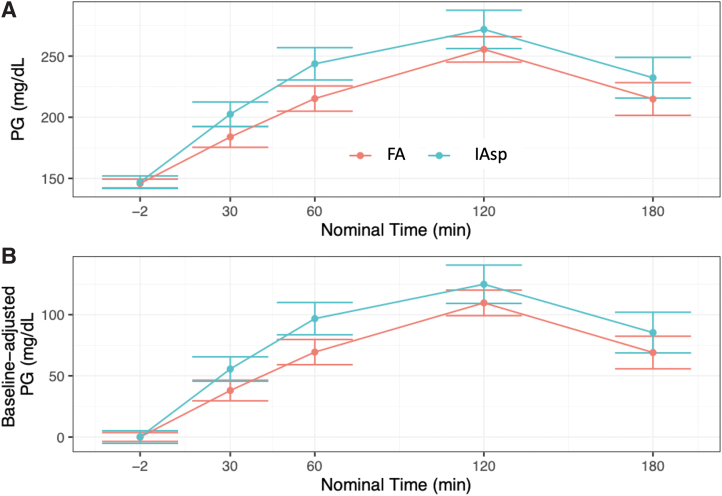
Actual **(A)** and baseline-adjusted **(B)** PG levels over time after infusion with faster aspart or IAsp after a standardized meal test after 6 weeks of use of faster aspart versus IAsp. Error bars represent standard error of the mean. PG, plasma glucose. Color images are available online.

Treatment with FA using the MiniMed 670G HCL led to a greater reduction in 1-h postprandial glucose increase compared with treatment with IAsp during the standardized mixed meal test. Change in glucose: [ETD (±SD); 95% CI]: 70.27 (±17.36) mg/dL (3.9 ± 1.0 mmol/L) with FA versus 98.42 (±17.36) mg/dL (5.5 ± 1.0 mmol/L) with IAsp (*P* = 0.008) (Figure 1 and Figure 2).

### Secondary endpoints

#### Glycemic excursion parameters

**FIG. 3.**
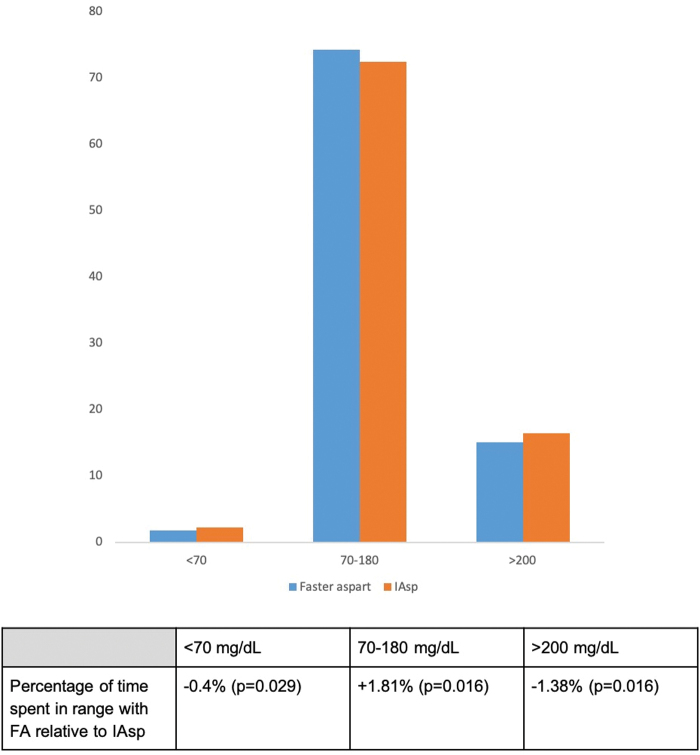
Percentage of time spent in range with faster aspart relative to IAsp. Color images are available online.

**FIG. 4.**
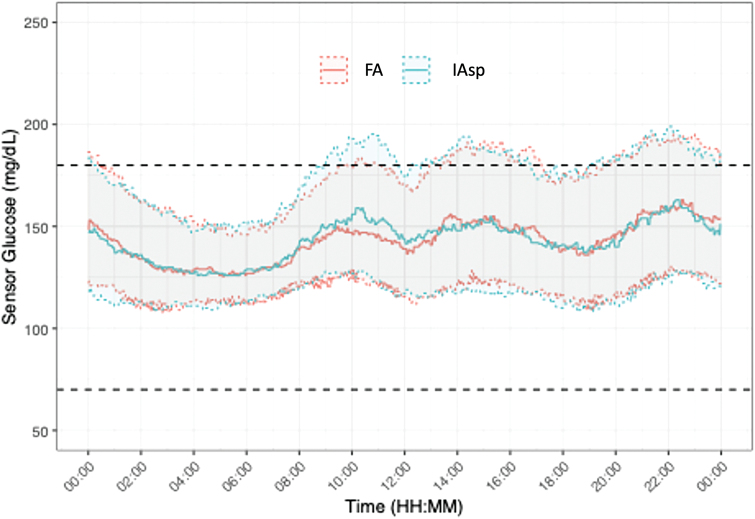
Median sensor glucose levels for 24 h. Continuous glucose monitor data covering weeks 3 through 7 of each treatment period aggregated across days and participants. Color images are available online.

No subjects spent any time below the 40 mg/dL (2.22 mmol/L) cutoff for severe hypoglycemia. The entire sample spent on average only 0.5% of time within the 40 to 54 mg/dL (2.22–3.0 mmol/L) range. Both of these ranges were dropped from further analysis. Time-in-range is depicted in Figure 3 and Figure 4. On average, patients spent 1.81% (*P* = 0.016) more time (roughly equivalent to 26 min per day) in the 70–180 mg/dL (3.89–9.99 mmol/L) range with FA than with IAsp. They spent 0.4% (*P* = 0.029) less time <70 mg/dL (3.89 mmol/L) with FA than with IAsp. Patients on FA spent 1.38% (*P* = 0.045) less time over 200 mg/dL (11.1 mmol/L) than with IAsp.

The change in glucose at 2 h was [ETD (±SD); 95% CI]: 110.05 (±20.92) mg/dL (6.1 ± 1.2 mmol/L) with FA versus 127.07 (±20.92) mg/dL (7.1 ± 1.2 mmol/L) with IAsp (*P* = 0.136).

In addition, level 1 time-above-range results (glucose of 181–250 mg/dL) were calculated. The patients on IAsp spent 0.45% more time in this range, but the difference was not statistically significant (*P* = 0.588).

#### Insulin-related parameters

Average total daily insulin dose did not show a significant change from study onset to end in either group (−1.00 for FA and −0.62 for IAsp, *P* = 0.957). There was a decrease in percentage bolus for FA, though this did not reach statistical significance (−2.97%, *P* = 0.276). There was no difference in either insulin-to-carbohydrate ratios or basal rates through the course of the study. Active insulin time was assigned and kept at 3 h. One patient's active insulin time was set at 4 h in error, and they were allowed to continue at that setting through the end of the study. Insulin-related parameters are detailed in Supplementary Table S2.

#### Pump-related parameters

Mean percentage of time spent in auto mode versus manual mode was 69.12% versus 30.88% with FA and 67.80% versus 31.91% for IAsp, and there was no statistically significant difference between these observations (*P* = 0.127).

#### HbA1c, fructosamine, and 1,5-anhydroglucitol levels

Comparisons of HbA1c (0.06% greater decrease with FA, *P* = 0.059), fructosamine (1.18 mmol/L greater decrease with FA, *P* = 0.968), and 1,5-anhydroglucitol levels (0.2 mcg/mL greater decrease with FA, *P* = 0.303) did not show a statistically significant difference.

#### Safety parameters

##### Infusion site reactions and occlusion events

A total of six infusion site reactions were reported and each agent had three events. A total of 29 occlusion events were recorded and there was no significant difference between FA (14 events) versus IAsp (15 events).

##### Reasons for switching to manual mode

Main reasons for switching to manual mode were high glucose levels, auto mode max, auto mode min, auto mode disabled by user, and pump suspended by user. Distribution of these between the two groups is detailed in Table 2.

**Table 2.** Distribution of Auto Mode Exit Events Between the Groups

Auto mode exit type	FA	IAsp
High serum glucose^a^	142	217
Auto mode max^b^	104	90
Auto mode min^c^	75	69
Auto mode disabled by user	85	86
Pump suspend by user	3	6
Unidentified	159	158

^a^ High SG: >300 mg/dL for 1 h or >250 mg/dL for 3 h).

^b^ Auto mode max: auto basal exceeded the 4-h time limit+safe basal (1.5 h).

^c^ Auto mode min: auto basal exceeded the 2.5 h time limit+safe basal (1.5 h).

FA, fast acting insulin aspart; IAsp, insulin aspart.

##### Hypoglycemia

No subjects spent any time <40 mg/dL (2.22 mmol/L). All study participants spent only 0.5% of time in level 2 hypoglycemia range (<54 mg/dL [3.0 mmol/L]). This hypoglycemia threshold is consistent with glucose concentrations that should be reported in clinical trials recommended by the joint position statement of the American Diabetes Association (ADA) and European Association for the Study of Diabetes (EASD) from 2017.^13^ There were no episodes of severe hypoglycemia.

## Discussion

This study demonstrated that the use of FA in the Medtronic 670G HCL system is safe and effective with a greater reduction in 1-h postmeal glucose and higher percentage time-in-range than IAsp.

This study is the first trial to evaluate the use of FA in the Medtronic 670G HCL system. Our study expands experience with FA use in other continuous subcutaneous insulin infusion settings.^6–8^

Other studies using FA given through CSII in the setting of a fixed basal rate have shown a postprandial glucose-lowering advantage for FA.^7^ In contrast, a study using FA in a closed loop setting through a different insulin delivery algorithm than the Medtronic 670G actually found higher postprandial glucose levels with FA and the time-in-range was similar to that of IAsp. It was postulated that this finding was due to the algorithm not being optimized for FA.^12^

In our study, there was a difference in the 1-h postmeal test glucose between the two insulins despite the insulin delivery algorithm in the 670G HCL system being “tuned” to the pharmacokinetic characteristics of IAsp and insulin lispro. This is consistent with the more rapid onset of action and earlier peak effect of this faster insulin. The 2-h postmeal test glucose was numerically lower but the difference did not meet statistical significance. It should be noted that the use of FA led to numerically lower glucose levels at each of the intervals tested with a statistically significant difference at 1-h postmeal test (Fig. 2a).

There may have been a greater difference in outcome if the pump algorithm had been adjusted for FA pharmacokinetics. A HCL system responds to glucose excursions after a standardized meal test, which may blunt differences between prandial insulins. As a result, the full effect of faster IAsp may not have been seen. It is possible that future HCL insulin administration algorithms will allow users to tailor settings to insulins with different pharmacodynamic characteristics.

In the 670G HCL system, basal dosing is determined every 5 min by an algorithm that takes into account the following factors: the magnitude and duration that the ambient glucose has varied from target, rate of rise or fall of the glucose, previously dosed insulin, active insulin time, and maximum auto mode basal that is recalculated every 24 h using total daily dose.^14^

A significant difference was seen on time-in-range (70–180 mg/dL [3.89–9.99 mmol/L]), and participants spent more time in the 70–180 mg/dL (3.89–9.99 mmol/L) range and less time in both the <70 mg/dL (<3.89 mmol/L) and >180 mg/dL (>9.99 mmol/L) ranges with FA. This may be a result of faster absorption of FA that may mimic normal insulin physiology more closely by attaining a more rapid prandial effect.^3^ In our study, a difference of 1.81% greater time spent in time-in-range with FA was seen. This would translate into 182 min more time in the desirable range per week. Time-in-range was defined per the clinical targets set by the international consensus on time-in-range.^15^

One of the glycemic target recommendations from ADA Standards of Medical Care in Diabetes, 2020, was that Time-in-Range (TIR) (percentage time glucose is in the range of 70–180 mg/dL) can be used for assessment of glycemic control, and time below and above target can be used to re-evaluate the treatment regimen.^16^ In our study, both treatment groups (FA and IAsp) met the ADA TIR target of >70% though FA had a significantly higher TIR and significantly lower time below and above the target range.

The findings of this study are in line with previous literature on the use of FA both in multiple dose insulin injection therapy and in continuous subcutaneous insulin infusion studies.^3–9^

The strengths of this study include its complete crossover design, which enabled subjects to serve as their own controls. A limitation of the study was the open-label design.

## Conclusion

In summary, we have demonstrated that the use of FA is safe and effective in the 670G HCL system. When compared with IAsp in the 670 g, the use of FA led to lower 1-h meal test glucose and more time spent in the 70–180 mg/dL (3.89–9.99 mmol/L) target range. FA is an appealing alternative for patients using the 670G HCL system.

## Supplementary Material

Supplemental data

Supplemental data
