# Supplementary material for: Fast Acting Insulin Aspart Compared with Insulin Aspart in the Medtronic 670G Hybrid Closed Loop System in Type 1 Diabetes: An Open Label Crossover Study
Source: Diabetes Technol Ther. 2021 Mar 22;23(4):286–92. doi: 10.1089/dia.2020.0500 (PMC7994433; doi:10.1089/dia.2020.0500)
Supplement: Supplemental data [file Supp_TableS1.docx]

Supplementary Table 1. Inclusion and exclusion criteria

| **Inclusion criteria** | **Exclusion criteria** |
| --- | --- |
| 1**.** Informed consent obtained before any trial-related activities. Trial-related activities are any procedures that are carried out as part of the trial, including activities to determine suitability for the trial  2.   Male or female, age ≥18 years at the time of signing informed consent  3.   Documented diagnoses of T1DM ≥1 year prior to the day of screening  4.   Using the Medtronic pump Minimed 670G for CSII in a basal-bolus regimen with a rapid acting insulin analogue for at least 30 days prior to screening and willing to continue using their personal Medtronic Minimed 670G and CSII for insulin treatment throughout the trial.  5.   Ability and willingness to use the same insulin infusion sets throughout the trial  6.   Using the same insulin for at least 30 days prior to screening  7.   HbA1c < 8.5% as assessed by local laboratory at screening  8.   BMI ≤ 35.0 kg/m2 at screening  9.   Ability and willingness to adhere to the protocol including performing SMPG profiles, attending visits, utilizing the auto mode feature of the pump for at least 80 % of the time during the study, and completing meal tests | 1.   Known or suspected hypersensitivity to trial products or related products   2.  Female who is pregnant, breast-feeding or intends to become pregnant or is of child-bearing potential and not using adequate contraceptive methods (adequate contraceptive measures as required by local regulation or practice)  3.   Participation in another clinical trial within 28 days before the screening visit. Note: clinical trials do not include non-interventional studies  4.   Anticipated significant change in lifestyle (e.g. eating, exercise or sleeping pattern) throughout the trial  5.   Any of the following: myocardial infarction, stroke, hospitalization for unstable angina or transient ischemic attack within the past 180 days prior to the day of screening   6.  Subjects classified as being in New York Heart Association (NYHA) Class IV at screening   7.   Planned coronary, carotid or peripheral artery revascularization known on the day of screening.   8.   Inadequately treated blood pressure defined as Grade 3 hypertension or higher (Systolic ≥180 mmHg or diastolic ≥110 mmHg) at screening  9. Impaired liver function, defined as ALT ≥ 2.5 times upper normal limit at screening  10. Renal impairment measured as estimated Glomerular Filtration Rate (eGFR) value of < 45 ml/min/1.73 m2   11. Anticipated initiation or change in concomitant medications (for more than 14 consecutive days) known to affect weight or glucose metabolism in the opinion of the Investigator   12. Proliferative retinopathy or maculopathy requiring acute treatment at the time of screening   13. History of hospitalization for ketoacidosis ≤180 days prior to the day of screening   14. Treatment with any medication for the indication of diabetes or obesity other than stated in the inclusion criteria within 30 days before screening   15. Presence of malignant neoplasms at the time of screening. Basal and squamous cell skin cancer and any carcinoma in-situ is allowed.   16. Reoccurring Severe hypoglycemia while on the Medtronic Minimed 670G in the investigators opinion   17. Any condition which, in the opinion of the Investigator, might jeopardize. Subject’s safety or compliance with the protocol |
