# Supplementary material for: Fast Acting Insulin Aspart Compared with Insulin Aspart in the Medtronic 670G Hybrid Closed Loop System in Type 1 Diabetes: An Open Label Crossover Study
Source: Diabetes Technol Ther. 2021 Mar 22;23(4):286–92. doi: 10.1089/dia.2020.0500 (PMC7994433; doi:10.1089/dia.2020.0500)
Supplement: Supplemental data [file Supp_TableS2.docx]

Supplementary Table 2. Insulin related parameters reported as mean (standard deviation)

| Measure | Baseline | Final | Change |
| --- | --- | --- | --- |
| **Average total daily dose (units)** | | | |
| All | 50.53 (28.00) | 49.72 (24.84) | -0.81 (10.52) |
| FA | 50.65 (28.59) | 49.65 (23.07) | -1.00 (10.49) |
| IAsp | 50.41 (27.78) | 49.78 (26.82) | -0.62 (10.68) |
| **Average total daily bolus (units)** | | | |
| All | 25.41 (13.65) | 24.51 (14.33) | -0.89 (5.66) |
| FA | 25.57 (13.65) | 24.16 (13.53) | -1.41 (5.69) |
| IAsp | 25.24 (13.84) | 24.86 (15.27) | -0.38 (5.65) |
| **Average total daily basal (units)** | | | |
| All | 25.61 (15.76) | 25.20 (12.70) | -0.41 (7.16) |
| FA | 25.08 (16.42) | 25.49 (12.08) | 0.41 (8.11) |
| IAsp | 26.14 (15.28) | 24.92 (13.44) | -1.22 (6.07) |
| **Percent bolus (%)** | | | |
| All | 50.43 (10.34) | 49.21 (10.31) | -1.22 (7.13) |
| FA | 51.26 (9.05) | 48.44 (10.33) | -2.81 (7.21) |
| IAsp | 49.61 (11.55) | 49.98 (10.37) | 0.37 (6.77) |
| **Percent basal (%)** | | | |
| All | 49.57 (10.34) | 50.79 (10.31) | 1.22 (7.13) |
| FA | 48.74 (9.05) | 51.56 (10.33) | 2.81 (7.21) |
| IAsp | 50.39 (11.55) | 50.02 (10.37) | -0.37 (6.77) |
| **Insulin : carbohydrate ratios** | | | |
| All | 8.79 (4.54) | 8.72 (4.92) | -0.07 (0.87) |
| FA | 8.92 (4.59) | 8.77 (4.74) | -0.15 (0.86) |
| IAsp | 8.65 (4.55) | 8.67 (5.16) | 0.02 (0.87) |
| **Basal rates (units / hour)** | | | |
| All | 1.02 (0.52) | 1.01 (0.52) | -0.01 (0.07) |
| FA | 1.02 (0.52) | 1.02 (0.52) | 0.00 (0.08) |
| IAsp | 1.02 (0.53) | 1.01 (0.53) | -0.02 (0.05) |
| **Active insulin time (hours)** | | | |
| All | 3.01 (0.16) | 3.00 (0.00) | -0.01 (0.16) |
| FA | 3.00 (0.20) | 3.00 (0.00) | 0.00 (0.20) |
| IAsp | 3.01 (0.08) | 3.00 (0.00) | -0.01 (0.08) |
